# Epigallocatechin-3-gallate improves cardiac hypertrophy and short-term memory deficits in a Williams-Beuren syndrome mouse model

**DOI:** 10.1371/journal.pone.0194476

**Published:** 2018-03-19

**Authors:** Paula Ortiz-Romero, Cristina Borralleras, Mònica Bosch-Morató, Biuse Guivernau, Guillermo Albericio, Francisco J. Muñoz, Luis A. Pérez-Jurado, Victoria Campuzano

**Affiliations:** 1 Departament de Ciències Experimentals i de la Salut, Universitat Pompeu Fabra, Barcelona, Spain; 2 Neurosciences Program, Institut Hospital del Mar d’Investigacions Mèdiques (IMIM), Barcelona, Spain; 3 Centro de Investigación Biomédica en Red de Enfermedades Raras (CIBERER), ISCIII, Barcelona, Spain; Maastricht University, NETHERLANDS

## Abstract

Williams-Beuren syndrome (WBS) is a neurodevelopmental disorder caused by a heterozygous deletion of 26–28 genes at chromosome band 7q11.23. The complete deletion (CD) mouse model mimics the most common deletion found in WBS patients and recapitulates most neurologic features of the disorder along with some cardiovascular manifestations leading to significant cardiac hypertrophy with increased cardiomyocytes’ size. Epigallocatechin-3-gallate (EGCG), the most abundant catechin found in green tea, has been associated with potential health benefits, both on cognition and cardiovascular phenotypes, through several mechanisms. We aimed to investigate the effects of green tea extracts on WBS-related phenotypes through a phase I clinical trial in mice. After feeding CD animals with green tea extracts dissolved in the drinking water, starting at three different time periods (prenatal, youth and adulthood), a set of behavioral tests and several anatomical, histological and molecular analyses were performed. Treatment resulted to be effective in the reduction of cardiac hypertrophy and was also able to ameliorate short-term memory deficits of CD mice. Taken together, these results suggest that EGCG might have a therapeutic and/or preventive role in the management of WBS.

## Introduction

Williams-Beuren syndrome (WBS) [OMIM 194050] is a neurodevelopmental disorder caused by the heterozygous deletion of 26–28 contiguous genes (1.55–1.83Mb) on chromosome 7q11.23 that affects 1/7500-1/20000 newborns [1,2]. WBS individuals present mild to moderate intellectual disability, with a mean intelligence quotient (IQ) of 55–60 [3–5]. The syndrome is characterized by a unique cognitive functioning including relatively preserved expressive language and facial processing abilities along with hypersociability towards strangers but dramatic deficits in spatial cognition [5–8]. Mainly due to Elastin (*ELN*) deficiency, WBS patients show a generalized arteriopathy characterized by hypertrophy of smooth muscle cells, increased number and disorganized lamellar structures, and fragmented elastic fibers [9]. The complete deletion (CD) mouse model captures several behavioral and cardiovascular defects that are seen in humans with WBS [10]. Other mouse models have been used to study the complex genotype-phenotype relationship associated with WBS, both assessing the role of genomic intervals and/or specific genes on the pathogenesis of WBS as well as defining possible therapeutic interventions [11–13].

In the last years, accumulating evidence supports that the consumption of green tea is associated with potential therapeutic effects on cancer, cardiovascular, inflammatory and neurological diseases [14,15]. In a mouse model of Down syndrome, green tea polyphenols rescued major features of the transgenic phenotype such as long-term memory and normalized *Bdnf* levels in hippocampus [16]. EGCG also improved cognitive function in a pilot study in Down syndrome individuals, resulting in an improvement in their quality of life [15]. The beneficial effects of EGCG have been mainly attributed to its potent antioxidant and radical scavenger functions. However, recent studies have suggested that, in addition to its antioxidant effects, its interaction with a wide range of cellular signaling pathways such as the phosphoinositide-3-kinase (PI3K) signaling pathway is also involved in the protective function of EGCG [17–20]. In heart, EGCG has been demonstrated to be a Ca^2+^ desensitizer, becoming a promising therapeutic agent for the hypertrophic cardiomyopathy caused by increased Ca^2+^ sensitivity of cardiac myofilaments [21].

In view of the neuro- and cardio- protective properties of EGCG in several diseases, we decided to explore its effects on CD mice. We show here that chronic administration of EGCG is able to rescue the hypertrophic cardiomyopathy possibly through increasing endogenous antioxidant activates by NRF2. In addition, EGCG treatment ameliorates short-term memory deficits of CD mice probably through normalization of *Bdnf* mRNA levels in the hippocampus of these mice.

## Materials and methods

### Ethics statement

Animal procedures were approved by the local Committee of Ethical Animal Experimentation (CEEA-PRBB; Protocol Number: VCU-14-1665) and by Generalitat of Catalonia (Protocol Number: DAAM-8101) in accordance with the guidelines of the European Communities Directive 86/609/EEC. The PRBB has Animal Welfare Assurance (#A5388-01, Institutional Animal Care and Use Committee approval date 05/08/2009), granted by the Office of Laboratory Animal Welfare (OLAW) of the US National Institutes of Health.

### Animals’ maintenance

CD mice (1.3Mb heterozygous deletion including from *Gtf2i* to *Fkbp6*) were obtained as previously described [10] and were crossed with Thy1-YFP transgenic mice (B6.Cg-Tg(Thy1-YFPH)2Jrs/J, Jackson Laboratory) [22] to label pyramidal neurons. All mice used were males and were maintained on 97% C57BL/6J background. Genomic DNA was extracted from mouse tail to determine the genotype of each mouse using MLPA and appropriate primers [12]. We used the following groups: WT-non treated n = 15; CD-non treated n = 10; WT-EGCG n = 13 and CD-EGCG n = 12. Animals were housed under standard conditions in a 12h dark/light cycle with access to food and water *ad libitum*. Finally, animals have been euthanized by a physical procedure of cervical dislocation.

### Epigallocatechin-3-gallate (EGCG) intake

EGCG was dissolved in drinking water and was prepared freshly from a green tea extract (Mega Green Tea Extract, decaffeinated, from Life Extension®, USA, EGCG content by HPLC 326,25 mg) every 48–60 hours. Following previous reports [15], we used a dose of 2.5–3 mg per day. The amount of drink per cage was quantified and we normalized it to the number of animals per cage (2 or 3) and to the number of hours that passed between each change (48 to 60).

Treatment was started in three different periods using different cohorts of mice: at 6 weeks old (young), at postnatal day 8 (P8 treatment) or treating the pregnant female at post-coitum (PC treatment). All treated animals, independently of the starting point, drank EGCG (2.5–3 mg per day) for 1 month until sacrifice at 8–12 weeks-old (young adult). The control group consisted of WT and CD mice drinking water without EGCG. All the experiments were carried out in the groups with different treatment starting points. Given the absence of significant differences in the results depending on the starting point, all data presented in this study correspond to the groups treated at adulthood (6 weeks).

As previously described, CD mice had significantly lower body weights compared to WT mice without influence of treatment (F_3,39_ = 8.361, *p* = 0.0002, S1A Fig). Daily EGCG consumption (ml per day) changed over time (repeated measures ANOVA, F_11_ = 4.893, *p*<0.001) but this significant effect was mainly due to the high uptake for day 2 (Bonferroni *post hoc* test). Consumption of EGCG or water was not significantly different between groups (main effect of treatment F_1,12_ = 0.049, *p* = 1.0) (S1B Fig).

### Behavioral tests

All the experiments were performed during the light phase of the dark/light cycle by researchers unaware of the different experimental groups. At the level of the task apparatus there was a constant illumination of about <10 lux. In all tests each apparatus was cleaned with a diluted ethanol solution after each mouse.

#### Novel object recognition (NOR) test

Object-recognition memory was assessed in a V-maze made out of black Plexiglas with two corridors at a 90° angle (30cm long x 4.5cm wide, and 15cm high walls) set at a 90° angle. On the first day, each mouse was individually habituated for 10 minutes to the maze in absence of objects. During the training session on the next day, two identical objects (A) were placed at the end of the corridors. The mouse was then placed in the middle of the maze and the total time spent exploring each object was recorded during 10 minutes. In the test session (10 minutes after the training session) the mouse was placed back in the maze, but a familiar object was replaced by a novel object (B). Time exploring each object was recorded again during 10 minutes. Object exploration was defined as time that the subject was in nose-to-object investigation. Throughout the experiments, objects were used in a counterbalanced manner to avoid possible spatial-location bias. To evaluate cognitive function a preference index was measured, defined as a ratio of the amount of time exploring any of the two objects in the training session or the novel object in the test session over the total time spent exploring both objects. Therefore, a preference index above 50% indicates novel object preference, below 50% familiar object preference, and 50% no preference [23,24].

#### Spontaneous alternation test

The spontaneous alternation test was conducted in a T-maze as previously described [11]. Briefly, mice were individually placed in the T-maze and allowed to freely choose between the two goals arms (A or B). A total of 15 free choices were annotated and the percentage of alternation during the free choice trials was calculated. The time to complete the 15 trials was also recorded and analyzed.

#### Social interaction test

We used the same previously described test [12], conducted in an open field. First, an empty wire cup-container was placed in the center of the arena. The subject mouse was allowed to explore the arena, and the amount of time sniffing the empty container was measured during 5 minutes. Next, an intruder mouse was hold in the container and, again, the amount of time nose to nose sniffing was measured during 5 minutes.

#### Marble-burying test

The test was conducted in a polycarbonate rat cage filled with bedding to a depth of 5cm and lightly tamped down. A regular pattern of 20 glass marbles (five rows of four marbles) was placed on the surface of the bedding prior to each test. An individual animal was placed in each cage. The number of buried marbles (>2/3 marble covered) was counted every five minutes during 20 minutes.

### Histological preparation

After cardiac perfusion with 1x PBS followed by 4% paraformaldehyde, brains and hearts were removed and postfixed in 4% paraformaldehyde for 24 hours at 4°C, in PBS for 24 hours at 4°C and, afterwards, crioprotected in 30% sucrose for 24 hours at 4°C. Finally, serial coronal sections (150μm) of brain were collected on a glass slide and directly mounted with Mowiol.

Hearts were paraffin embedded and serial coronal sections (8μm) were collected on a glass slide. Sections were stained with a regular hematoxylin-eosin protocol. Similar sections were used to perform DHE staining (Invitrogen). Sections were incubated with Triton 0.3% for 10 minutes and with a 5μM DHE solution for 90 minutes at 37°C. Sections were mounted with Mowiol over the same glass slide.

### Imaging

For morphological analysis of the stratum radiatum (SR) and stratum oriens (SO), 1360x1024 images of CA1 hippocampus were obtained with an Olympus DP71 camera attached to an Olympus BX51 microscopy with an Olympus U-RFL-T source of fluorescence at 4x magnification. Measures from six hippocampal sections per animal were averaged. Representative images of each animal group are shown in S2A Fig. For spine density and spine length analyses we obtained 1024x1024 pixel confocal fluorescent image stacks from coronal tissue sections of using a TCS SP2 LEICA confocal microscope. Measurements were performed according to a previously published method [10,12]. Briefly, basal proximal (30–120 μm from soma) 15–30 μm dendritic segments of randomly selected neurons were selected for the analysis of spine density. The number of spines was counted using ImageJ Cell Counter. Spines located on the top or bottom surfaces of the dendrites were not counted. Spine density was calculated by dividing the total spine count by the length of dendrite analyzed. Spine length was measured from the base of the spine neck to the end of the head of the spine (S2B Fig).

Images of hematoxylin-eosin stained heart samples were obtained using visible light with an Olympus DP71 camera attached to an Olympus MVX10 MacroView Upright Microscope (zoom factor 1,25) or to an Olympus BX51 microscopy with an Olympus U-RFL-T source of fluorescence (10X and 20X magnification). Cardiomyocytes’ areas and fluorescence intensity (DHE stained samples) were measured using ImageJ software.

### RNA preparation and gene expression quantification

RNA was extracted from hippocampal or cardiac tissues of adult mice using TRIZOL reagent (Invitrogen) according to the manufacturer’s instructions. cDNA was prepared from 1μg of total RNA using random hexamers and SuperScript II RNase H reverse transcriptase (Invitrogen). The expression of *Ryr2*, *Ryr3*, *Trpc3*, *Trpc6*, *Cacna1c* and *Atp2a2* in hippocampal tissue was evaluated by RNAseq. The expression of *Bdnf*, *Pik3r1*, *Ncf1*, *Ncf2*, *Rac2*, *Cyba*, *Nos3*, *Hsp90*, *Cav1*, *Cacna1c*, *Atp2a2*, *Nqo1*, *Limk1* and *Rps28* was evaluated by quantitative real-time PCR (qRT-PCR) and/or semi-quantitative RT-PCR as previously described [12] using the appropriate primers (S1 Table). Each PCR was made with triplicates and at least from three different RTs.

### Mouse primary cultures

Primary cultures were carried out following a procedure approved by the Ethics Committee of the Institut Municipal d’Investigacions Mèdiques-Universitat Pompeu Fabra (EC-IMIM-UPF).

#### Cardiomyocytes

One- to three-day-old neonatal mice were euthanized by cervical dislocation. Hearts were removed and kept in ice-cold Hanks’ balanced salt solution (HBSS) without Ca^2+^ and Mg^2+^ (Life Technologies). Hearts were washed thrice with the same HBSS and minced into small fragments. Cells were dissociated for 10 min with 50μl 0.25% wt/vol trypsin and suspended in 1ml DMEM-20%FBS. The suspended cells were divided in two equal parts and plated in 24-well plates, incubated under standard conditions with medium changing every two days. Primary cardiomyocytes were used at 8–10 days of culture (DIV).

#### Hippocampal neurons

Briefly, hippocampal neurons were isolated from 18-day-old mouse embryos. Brain was removed and hippocampus was aseptically dissected in ice-cold HBSS (Life Technologies) supplemented with 4.5g/L glucose (Sigma-Aldrich) and trypsinized for 17 min at 37°C. In order to eliminate rests of the trypsinization medium and to disaggregate the cells, the cell solution was washed thrice in HBSS+glucose and mechanically dissociated. Then the cells were then seeded on DMEM (Life Technologies) plus 10% horse serum (Life Technologies) onto 1% poly-D-Lysine (Sigma-Aldrich, USA) coated plates. Once the neurons were attached to the polylysinated wells (2h), the seeding medium was removed and Neurobasal medium (Life Technologies) was added containing 2% B27 supplement (Gibco BRL), 1% GlutaMAX (Life Technologies) and 1% Penicillin/Streptomycin. On 3 DIV, cells were treated with 2μM 1-β-D-arabinofuranosylcytosine (AraC; Sigma) for 24h to eliminate glia. Primary hippocampal neurons were used at 10 DIV.

### Immunocytochemistry

Cells were washed with PBS, fixed with 100% methanol at -21°C for 15min, incubated for 30min at 37°C with blocking solution (0.1% Triton-X, 10% FBS in PBS) and incubated overnight at 4°C with the primary antibody [NRF2 (1:150, Santa Cruz Biotechnology), TRPC3 (1:500, Sigma) and MAP2 (1:500, Sigma)]. After washing, secondary antibody (1:1000, Invitrogen) was diluted in secondary blocking solution (13% FBS in PBS) and incubated for 1h at room temperature in the dark. Finally cells were mounted on a glass slide in a 7μl drop of mounting solution (ProLong™ Gold antifade reagent with DAPI, Invitrogen).

Images were obtained at 40X magnification with an Olympus DP71 camera attached to an Olympus BX51 microscopy with an Olympus U-RFL-T source of fluorescence and fluorescence intensity was measured with ImageJ software.

### Intracellular calcium analysis

Hippocampal neurons were incubated with 250μL Fura-2 AM (5μM; Life Technologies) and 0.02% pluronic acid for 40min at room temperature and washed thoroughly with isotonic solution containing 2.5mM KCl, 140mM NaCl, 1.2mM CaCl2, 0.5mM MgCl_2_, 5mM glucose and 10mM HEPES (305mOsm, pH 7.4). Calcium analysis was performed with a custom-made recording chamber on an inverted microscope at room temperature. The frequency, amplitude and duration of the calcium peaks were measured with excitation at 340 and 380nm and emission at 510nm. Fluorescence was recorded every 5s using a digital camera controlled by AquaCosmos software. Data were corrected for the basal individual fluorescence measured prior to stimulation.

### Western blot

Cardiac tissue was homogenized using tissue protein extraction buffer (0.15M NaCl, 1% Triton-X, 10% glycerol 85%, 0.001M EDTA, 0.05M Tris; pH 7.4) containing protease inhibitors (cOmplete™ ULTRA Tablets Mini, Roche) and sonicated. Protein concentrations were determined with Bradford reagent (Bio-Rad protein assay). Proteins (100μg) were separated in polyacrylamide gels (4–20% Mini-PROTEAN® TGX™ Gel, Bio-Rad) and transferred to polyvinylidene difluoride membranes (Millipore). Primary RyR2 antibody (1:1000, Sigma-Aldrich) was incubated overnight at 4°C, using actin (1:3000, Sigma-Aldrich) as a gel-loading control. Secondary antibodies (1:6000, Rockland) were incubated 1h at room temperature in the dark, and blots were developed using Odyssey® Imaging System (LI-COR Biosciences). Images were quantified with ImageJ software.

### Statistical analysis

All data are presented as means ± SEM. Unpaired *t-*test with Welch’s correction and one-way, two-way or three-way ANOVA with Bonferroni or Dunnett’s *post hoc* test, were used when needed. Novel object recognition data were analyzed by one-sample *t*-tests to examine whether object exploration times were different from the 50% chance level. Values were considered significant when *p<*0.05. GraphPad Prism software was used for all statistical tests and graphs.

## Results

### EGCG improves short-term memory but not working memory in CD mice

**Fig 1:**
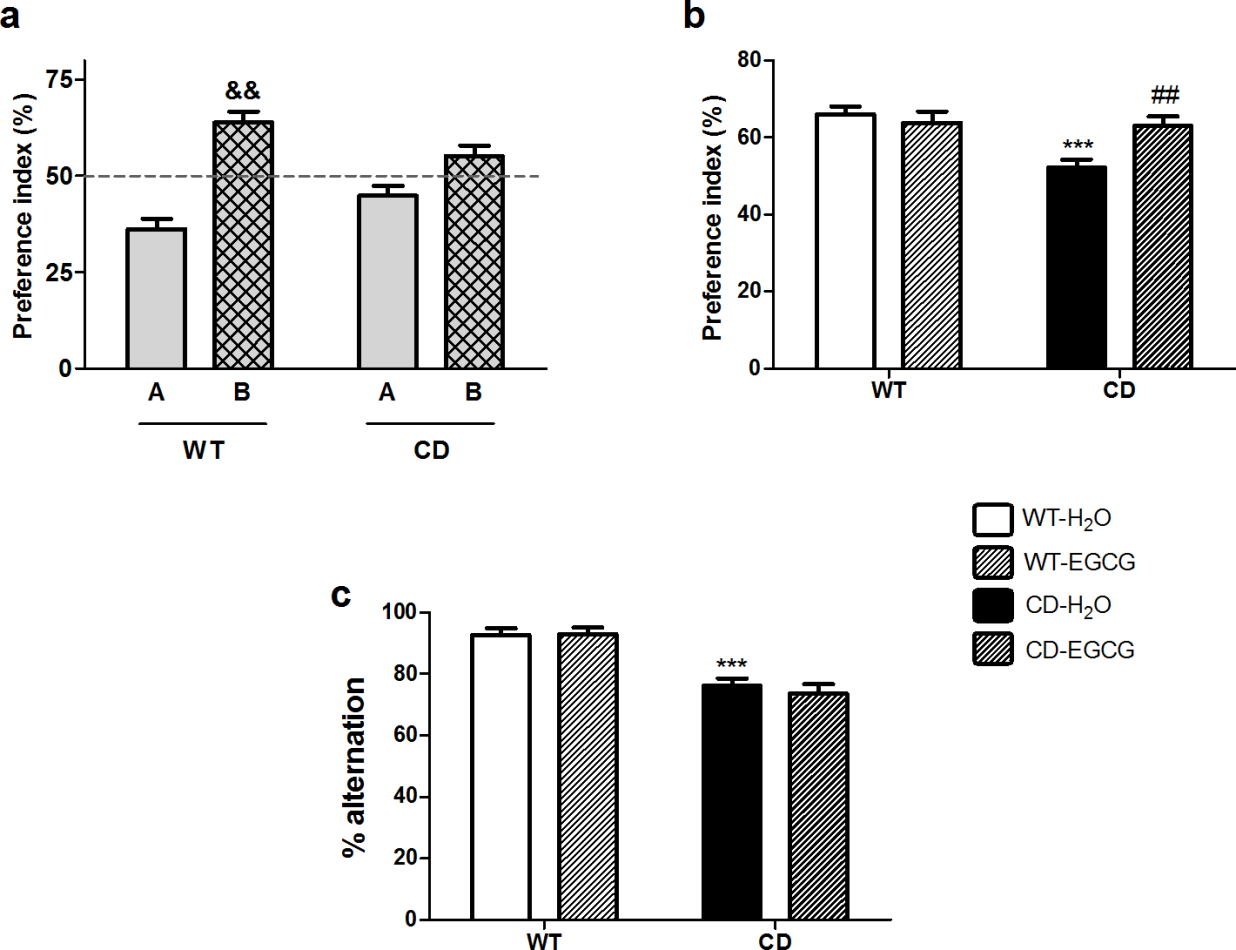
EGCG rescues short-term but not working memory deficits in CD mice. (a) Representation of the preference index for the novel object in the test session in animals fed with water (H_2_O). WT animals displayed preference for the novel object (*t*_7_ = 7.951, *p* = 0.0002). In contrast, CD mice were unable to discriminate between the two objects (*t*_7_ = 0.979, *p* = 0.360). *p* values are shown with ampersand indicating values that are significantly different in one-sample *t*-test compared with 50% chance level. Grey bar, familiar object; grid bar, novel object. (b) Representation of the preference index for the novel object in the test session. EGCG treatment increased exploration time of the novel object of CD mice respect to water fed CD mice (*p*≤0.01, Bonferroni *post hoc* test). EGCG treatment had not effect in WT mice (*p*>0.05, Bonferroni *post hoc* test). n = 7–8 per group. (c) Percentage of alternation (number of alternations/total number of possible alternation·100) in the spontaneous alternation test. A two-way ANOVA indicated a significant effect of genotype (F_1,39_ = 47.76, *p*<0.0001) but no effect of treatment (F_1,39_ = 0.1464, *p* = 0.7041). n = 8–13 per group. *p* values are shown with asterisks (genotype effect) or hashes (treatment effect) indicating values that are significantly different in a two-way ANOVA with Bonferroni’s *post hoc* test (^##^*p*<0.01,***p<0.001) White, WT; Black, CD. Plain, water; Striped, EGCG. Data are presented as the mean ± SEM.

Short-term memory of CD mice was explored with the Novel Object Recognition (NOR) test using an interval of 10 minutes between the training and test sessions. Animals of both genotypes explored equally the two identical objects (A+A) during training (S3A Fig). In the test session, when a familiar object was replaced by a novel one (A+B), WT animals displayed preference for the novel object (B) that was significantly different from the 50% chance level (one-sample *t*-test t_7_ = 7.951, *p* = 0.0002). In contrast, CD mice were unable to discriminate between the objects, with no significant difference from chance levels (*t*_7_ = 0.979, *p* = 0.360) (Fig 1A). Interestingly, we could observe a significant interaction between genotype and treatment (two- way ANOVA, F_1,26_ = 7.419, *p* = 0.0116). CD mice treated with EGCG displayed increased exploration of the novel object which was significantly different from CD control mice (*p*≤0.01, Bonferroni *post hoc* test) (Fig 1B). EGCG treatment in WT mice did not have any effect on their performance in the task (*p*>0.05, Bonferroni *post hoc* test) (Fig 1B). Regarding the total object exploration time, a significant main effect of genotype was observed (two-way ANOVA F_1,26_ = 12.68, *p* = 0.0015), with CD mice showing significantly more exploratory activity than WT mice (*p*<0.05, Bonferroni *post hoc* test) (S3B Fig).

Spatial working memory in CD mice was determined by measuring the rate of spontaneous alternation (visiting each arm in turn) in the spontaneous alternation test. CD animals presented a significant decrease in the alternation rate when compared to WT animals (F_1,39_ = 47.76, *p*<0.0001). Treatment with EGCG did not improve the performance of CD mice in this test (F_1,39_ = 0.1467, *p* = 0.7041) (Fig 1C). Additionally, the total time to complete 15 trials did not differ between genotypes or treatment groups (S3C Fig).

### EGCG does not influence sociability or anxiety-related behavior in CD mice

**Fig 2:**
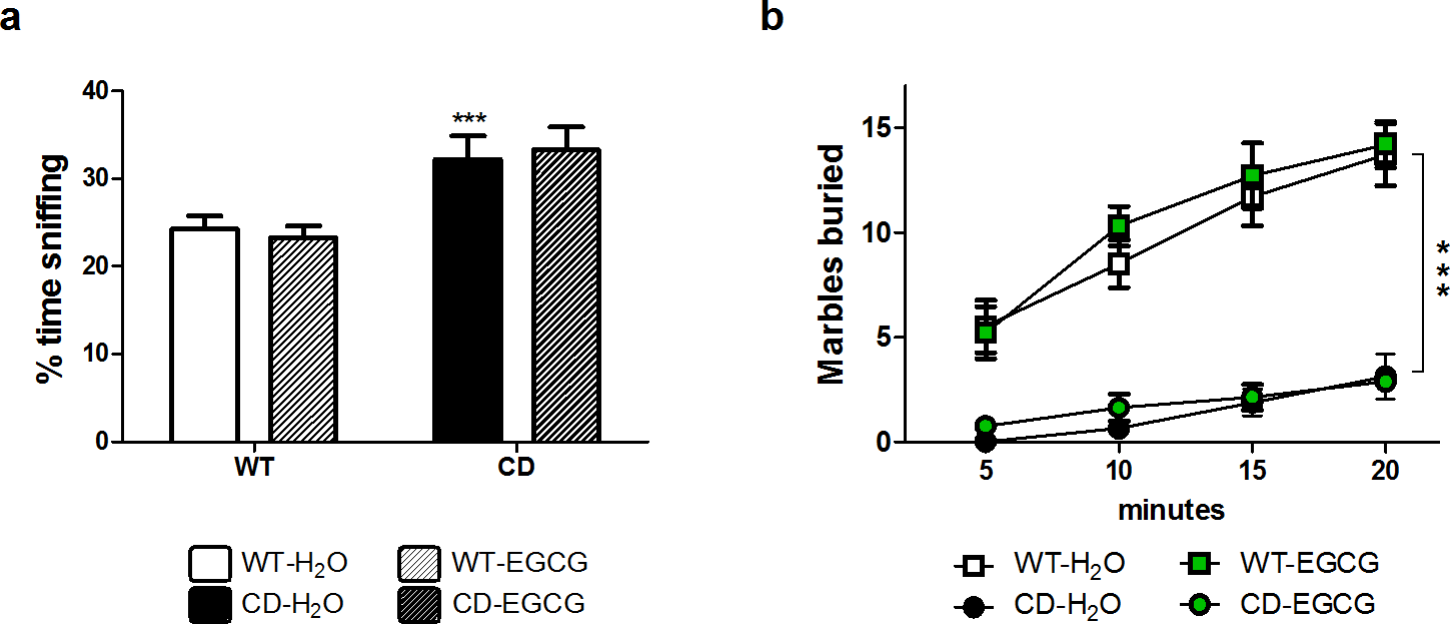
EGCG does not influence sociability or anxiety-related behavior in CD mice. (a) In a direct social test, CD mice fed with green tea behaved in the same way that water fed CD mice towards an intruder mice. A two-way ANOVA indicated a significant main effect of genotype (F_1,37_ = 19.86, *p*<0.0001), without effect of treatment (F_1,37_ = 0.00044, *p* = 0.9834). n = 7–15 per group. White, WT; Black, CD. Plain, water; Striped, EGCG. Data are presented as the mean ± SEM. (b) Anxiety-like behavior was evaluated in the marble-burying test. A three-way ANOVA revealed no interaction between genotype, treatment and time (F_3,128_ = 1.380, *p* = 0.252), with a significant main effect of genotype (F_1,128_ = 306.684, *p*<0.0001) and time (F_3,128_ = 17.170, *p*<0.0001) but no effect of treatment (F_1,128_ = 0.182, *p* = 0.670). n = 8–10 per group. Squares, WT; Circles, CD. White and black, water; Green, EGCG fed mice. *p* values are shown with asterisks (genotype effect) indicating values that are significantly different in a two-way ANOVA with Bonferroni *post hoc* test (***p<0.001).

In previous studies, CD mice showed a hypersociable phenotype resembling the overfriendly and outgoing behavior towards strangers present in WBS individuals [10,12]. In addition, the performance of CD mice in the marble-burying test was completely different from WT, suggesting alterations related to anxiety-like behavior [12]. We evaluated whether EGCG had any effect on sociability or anxiety-like behavior. As shown in Fig 2A, when comparing the time passed in the container with the intruder mouse among groups, we observed a main effect of genotype (F_1,37_ = 19.86, *p*<0.0001) but no effect of treatment (F_1,37_ = 0.00044, *p* = 0.9834). Regarding the marble-burying test, CD mice fed with EGCG performed as different from WT as mutant mice fed with water (Fig 2B). Therefore, EGCG treatment did not influence sociability or anxiety-related behavior in CD mice.

### EGCG does not affect neuroanatomical features of CD mice

**Fig 3:**
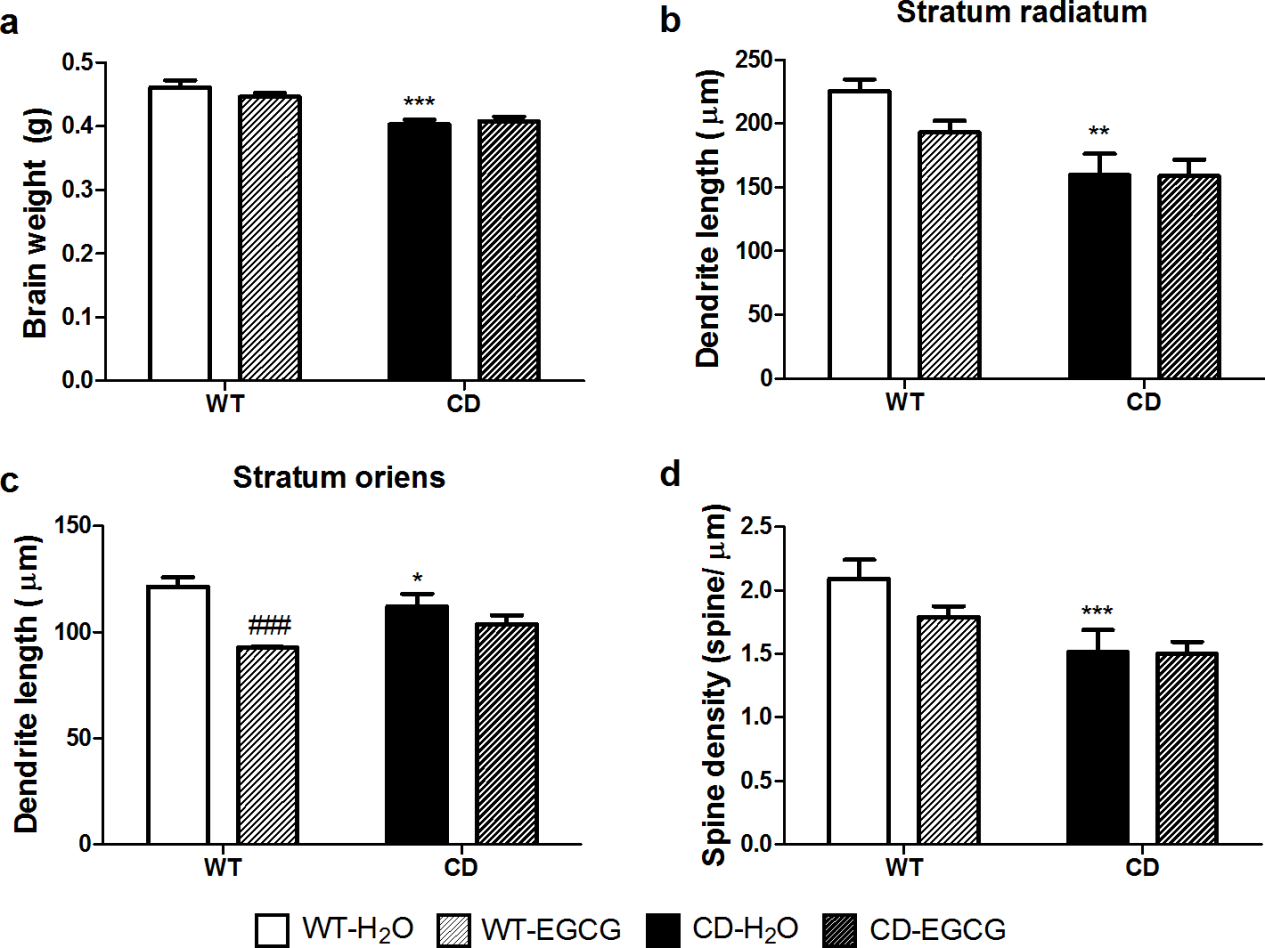
EGCG does not influence brain abnormalities present in CD mice. (a) Brain weight of water or EGCG fed CD mice was significantly reduced when compared to WT mice. A two-way ANOVA indicated a significant effect of genotype (F_1,37_ = 40.72, *p*<0.0001) but no effect of treatment (F_1,37_ = 0.3766, *p* = 0.5432). (b) Dendrite length of CA1 neurons was measured in stratum radiatum (SR). A two-way ANOVA indicated a significant effect of genotype (F_1,37_ = 15.98, *p* = 0.0003) but not effect of treatment (F_1,37_ = 1.728, *p* = 0.1968) (c) Dendrite length of CA1 neurons was measured in stratum oriens (SO). A two-way ANOVA indicated a deleterious effect of treatment in the case of WT mice (F_1,37_ = 22.92, *p*<0.0001 with *p*<0.001 Bonferroni *post hoc* test). (d) EGCG treatment did not increase the number of spines present in apical dendrites of CD neurons. A two-way ANOVA indicated a significant effect of genotype (F_1,37_ = 13.13, *p* = 0.0009) without treatment effect (F_1,37_ = 1.722, *p* = 0.1976). n = 8–13 mice per group. *p* values are shown with asterisks (genotype) or hashes (treatment) indicating values that are significantly different (two-way ANOVA with Bonferroni *post hoc* test). *,^#^*p*<0.05; **,^##^*p*<0.01; ***,^###^*p*<0.001. White, WT; Black, CD. Plain, water; Striped, EGCG fed mice. Data are presented as the mean ± SEM.

As we previously described [10], brain weight of water fed CD mice was significantly reduced when compared to water fed WT mice (12.74%). Brains from CD mice fed with EGCG were not different from CD control mice (effect of genotype, F_1,37_ = 40.72, *p*<0.0001, effect of treatment F_1,37_ = 0.3766, *p* = 0.5432) (Fig 3A). Therefore, EGCG did not induce differences in the total brain weight in CD mice.

We then focused on the hippocampus, the main brain structure involved in learning and memory. Previous studies in CD mice showed a reduction in dendritic length in both stratum radiatum (SR) and stratum oriens (SO) together with a significant reduction of about 17% in spine density in apical proximal dendrites of CA1 region of the hippocampus [10,12]. We could not appreciate any effect of EGCG on dendrite length (*p*>0.05, Bonferroni *post hoc* test) neither in spine density of treated CD mice (Fig 3B–3D and S2 Fig). Unexpectedly, we observed deleterious effects in WT mice treated with EGCG. These mice presented a significant reduction in the length of dendrites in SO (*p*<0.001, Bonferroni *post hoc* test) when compared to water fed WT animals (Fig 3B–3D and S2 Fig).

### EGCG treatment restores *Bdnf* mRNA expression levels in the hippocampus of CD mice

**Fig 4:**
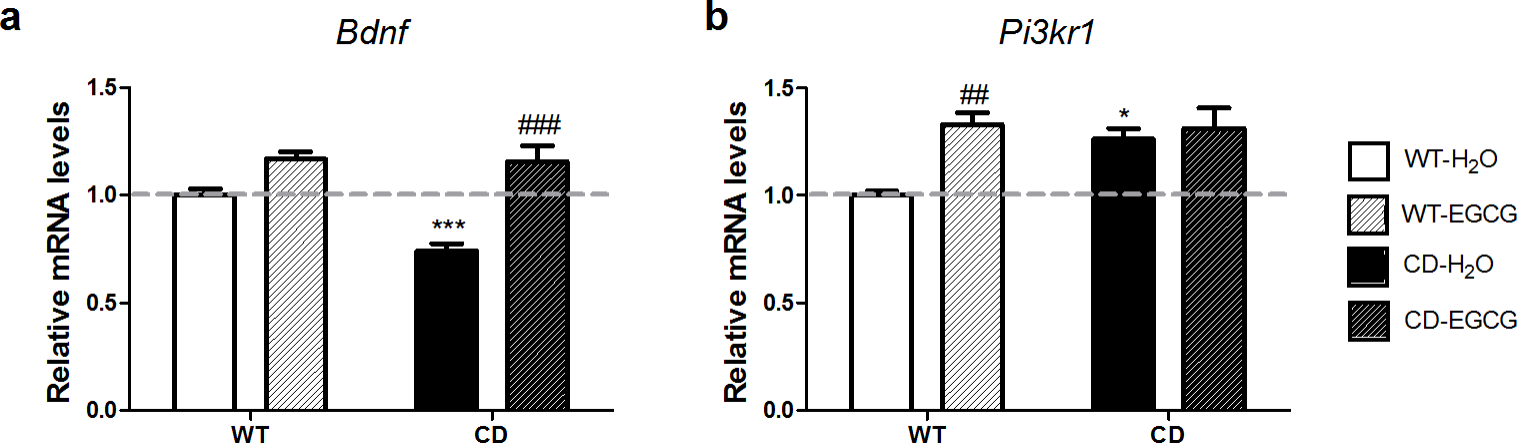
Effect of EGCG on hippocampal synaptic plasticity markers. (a) EGCG treatment normalized *Bdnf* mRNA levels in CD animals. A two-way ANOVA indicated a significant interaction between genotypes and treatment (F_1,38_ = 5.512, *p* = 0.0242), with a significant effect of genotype (F_1,38_ = 6.599, *p* = 0.0143) and treatment (F_1,38_ = 30.47, *p*<0.0001). (b) EGCG treatment did not normalize the high *Pik3r1* mRNA levels observed in the hippocampus of CD mice. A two-way ANOVA indicated a significant effect of treatment (F_1,24_ = 7.406, *p* = 0.0119), since EGCG treatment upregulated *Pik3r1* mRNA levels in WT animals. n = 6–13 per group. *p* values are shown with asterisks (genotype) or hashes (treatment) indicating values that are significantly different (two-way ANOVA with Bonferroni *post hoc* test). *,^#^*p*<0.05; **,^##^*p*<0.01; ***,^###^*p*<0.001. White, WT; Black, CD. Plain, water; Striped, EGCG. Data are presented as the mean ± SEM.

Brain-derived neurotrophic factor (BDNF) is a key signaling molecule for hippocampal synaptic plasticity and spatial memory. Several studies have supported the role of EGCG in the expression or activity of BDNF [16,25,26]. CD mice have significantly lower levels of *Bdnf* mRNA and protein in the hippocampus [11,12]. Positively, this defect was completely corrected by EGCG treatment, since CD mice fed with EGCG reached *Bdnf* levels similar to WT animals (Fig 4A).

The interaction of BDNF with its receptor activates the PI3K-Akt-mTor signaling pathway [27]. This pathway has been suggested to be deregulated in WBS, since the regulatory subunit of the PI3K (*Pik3r1*) is a direct target of TFII-I, a general transcription factor encoded by *GTF2I* included in the deleted region in WBS [12,28]. In fact, CD mice presented upregulated levels of *Pik3r1* in the hippocampus independently of *Bdnf* levels [12]. As expected, EGCG treatment in WT mice significantly increased the levels of *Pik3r1* in these mice, probably through BDNF-activation. However, in agreement with previous report [12], *Pik3r1* levels in CD mice fed with EGCG remained as high as in CD mice fed with water (Fig 4B).

### EGCG treatment prevents cardiac hypertrophy observed in CD mice

**Fig 5:**
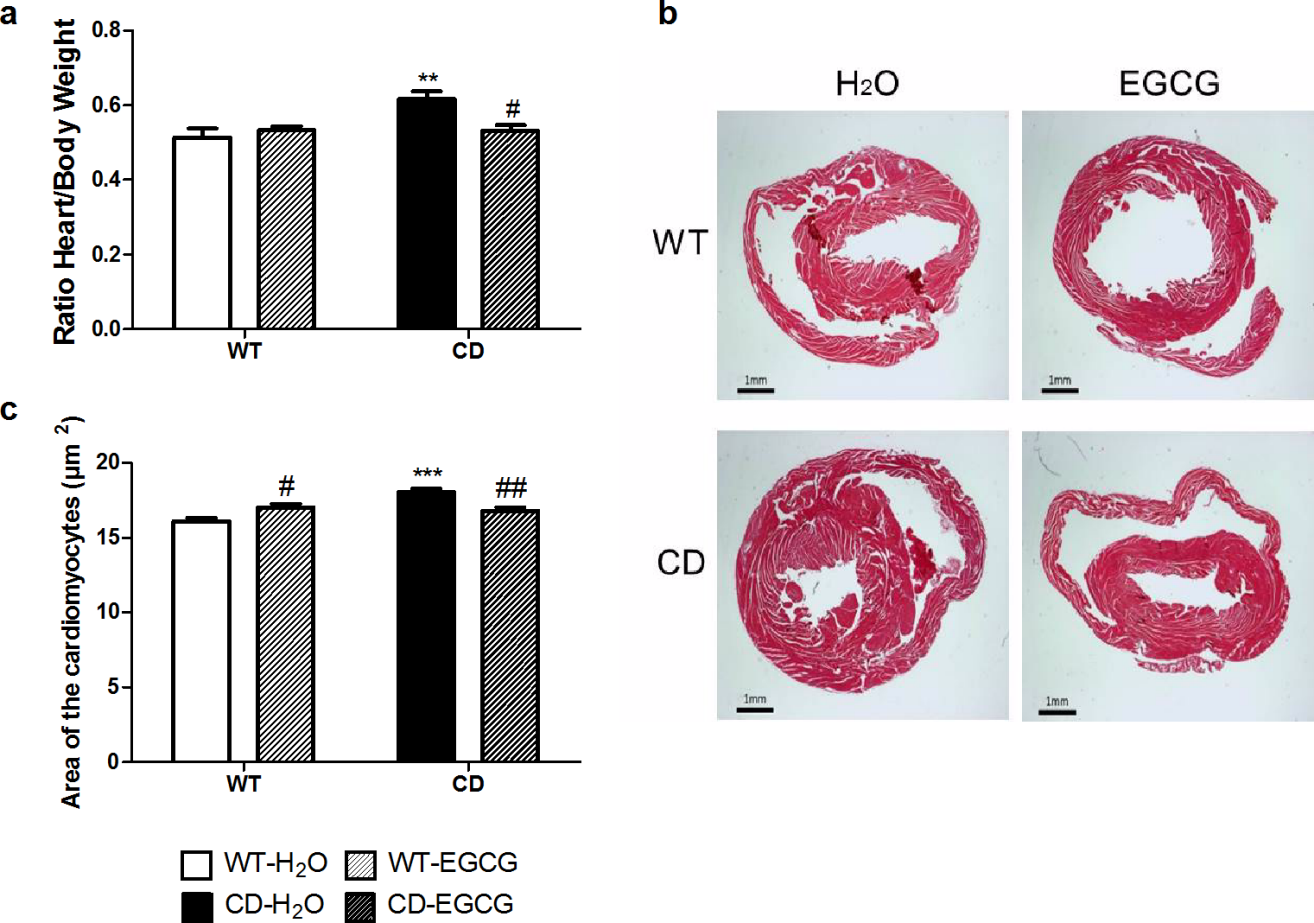
EGCG treatment prevents the cardiac hypertrophy observed in CD mice. (a) Comparison of the heart/body weight ratios obtained from each genotype showed that cardiac hypertrophy present in CD mice is not observed after EGCG treatment. A two-way ANOVA indicated a significant interaction between genotypes and treatment (F_1,46_ = 7.237, *p* = 0.0099), with a significant effect of genotype (F_1,46_ = 6.751, *p =* 0.0125) but with treatment effect only in CD mice (*p*<0.01, Bonferroni *post hoc* test). n = 10–15 mice per group. (b) Representative images of hematoxylin-eosin stained transverse sections of heart samples. We can appreciate the thickening of the left ventricular wall of the control CD animals. (c) CD mice cardiomyocytes are significantly larger than in WT animals, while CD animals treated with EGCG show normal cell size. A two-way ANOVA indicated a significant interaction between genotypes and treatment (F_1,12_ = 24.05, *p* = 0.0004), with a significant effect of genotype (F_1,12_ = 14.15, *p* = 0.0027) and treatment effect in CDs (*p*<0.01, Bonferroni *post hoc* test) but also in WTs (*p*<0.05, Bonferroni *post hoc* test). n = 4 mice/group. *p* values are shown with asterisks (genotype) or hashes (treatment) indicating values that are significantly different (Two-way ANOVA with Bonferroni *post hoc* test). *,^#^*p*<0.05; **,^##^*p*<0.01; ***,^###^*p*<0.001. White, WT; Black, CD. Plain, water; Striped, EGCG. Data are presented as the mean ± SEM.

The cardiovascular phenotype observed in WBS patients is mainly caused by elastin deficiency and also has been linked to elevated angiotensin II (AngII) levels and secondary NADPH-oxidase activation with increased *NCF1* levels [29,30]. CD mice show a slight increase of arterial blood pressure and engrossment of the aortic wall as well as a significant cardiac hypertrophy that correlates with increased size of cardiomyocytes [10]. With the aim of evaluating whether EGCG treatment improves cardiac hypertrophy, we compared the hearts’ weight and the cardiomyocytes’ area between treated and untreated CD mice. Consistent with previous results, a significant cardiac hypertrophy was observed in CD animals in comparison with the WT (*p*<0.01, Bonferroni *post hoc* test) (Fig 5A and 5B). A two-way ANOVA indicated a significant interaction between genotypes and treatment (F_1,46_ = 7.237, *p* = 0.0099), CD animals treated with EGCG showed a significant reduction of hearts’ weight compared with control CD mice (*p*<0.05, Bonferroni *post hoc* test) (Fig 5A and 5B). This normalization in weight correlated with the normalization of the cardiomyocytes’ size; while CD cardiomyocytes were larger than WT cardiomyocytes (*p*<0.001, Bonferroni *post hoc* test), after EGCG treatment the size of the cardiomyocytes in CD-treated mice was significantly different of control CD mice (*p*<0.01, Bonferroni *post hoc* test) (Fig 5C).

### Impaired cellular calcium homeostasis in CD mice

**Fig 6:**
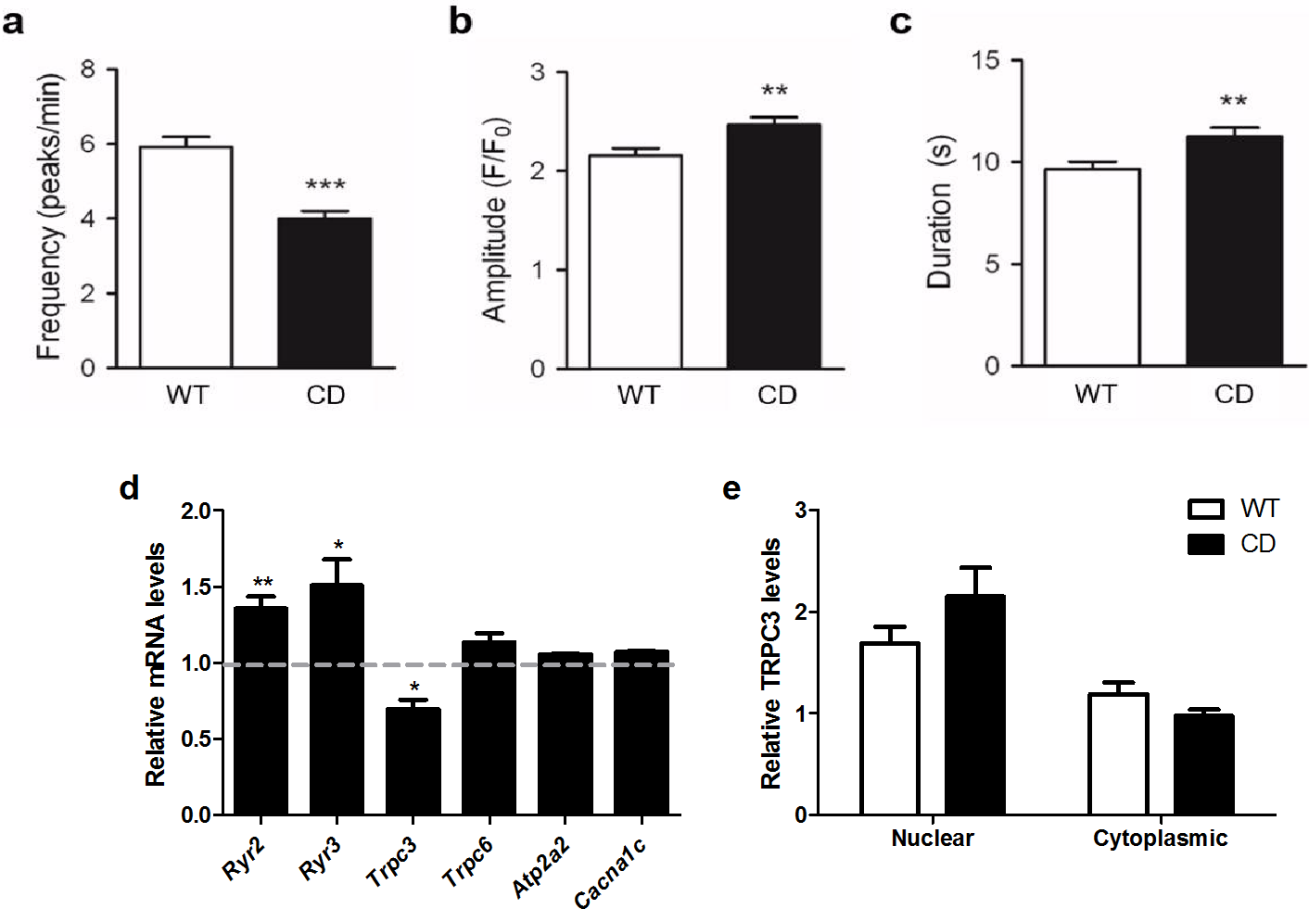
Impaired cellular calcium homeostasis in CD mice. (a) The mean frequency of calcium oscillations in CD hippocampal neurons was significantly lower (*t*_200_ = 5.526, *p*<0.0001) when compared to the mean frequency of WT neurons n = 103–130 cells per genotype. (b) Amplitude (*p* = 0.0028) and (c) duration (*p* = 0.0063) of calcium transients were significantly higher in CD neurons when compared to WT neurons. (d) Differences in hippocampal mRNA expression genes related to Ca^2+^ signaling levels of *Trpc3* were slightly lower in CD mice (*p* = 0.045), while *Ryr2* and *Ryr3* levels were significantly higher in CD mice when compared to WT mice (*p* = 0.0017 and *p* = 0.0071, respectively). No significant differences in mRNA expression of other genes related to Ca^2+^ signaling (*Cacna1c*, *Atp2a2* and *Trpc6*) were found. (e) TRPC3 subcellular localization in CD mice primary cultures of hippocampal neurons is not significantly altered compared with WT cells (F_1,77_ = 0.3922, *p* = 0.5331). n = 16–23 cells per genotype. *p* values are shown with asterisks (genotype) indicating values that are significantly different (two-way ANOVA with Bonferroni *post hoc* test or unpaired *t*-test with Welch’s correction). *p<0.05; **p<0.01; ***p<0.001. White, WT; Black, CD. Plain, water. Data are presented as the mean ± SEM.

Memory processing and cardiomyocytes function requires tightly controlled signaling cascades, many of which are dependent upon intracellular calcium (Ca^2+^) [31,32]. We previously demonstrated that long-term potentiation (LTP) elicited by theta burst stimulation (TBS) is significantly impaired in hippocampal field CA1 of CD animals [11]. Therefore, we decided to characterize this phenomenon by measuring intracellular calcium concentration in hippocampal primary cultures of CD mice. Both WT and CD hippocampal cultures exhibited highly synchronized calcium oscillations. The mean frequency of calcium oscillations in CD neurons was significantly lower (*t*_200_ = 5.526 *p*<0.0001, unpaired *t* test with Welch’s correction) when compared to the mean frequency of WT neurons (CD: 3.99±0.21 peaks/min vs. WT: 5.91±0.28 peaks/min) (Fig 6A). Both amplitude and duration of calcium transients were significantly higher in CD neurons when compared to WT neurons (Fig 6B and 6C). The analysis of hippocampal mRNA levels of genes related to Ca^2+^ signaling showed slightly lower amount of *Trpc3* (*p* = 0.045) but no differences for *Trpc6*. Ryanodine receptors (*Ryr2* and *Ryr3*) were significantly higher in CD mice when compared to WT mice (*p* = 0.0017 and *p* = 0.0071, respectively). No significant differences in mRNA expression of other calcium-related genes such as *Cacna1c* and *Atp2a2* were found (Fig 6D). TRPC3 has been previously related to altered calcium homeostasis of WBS patients through TFII-I [33–35]. TRPC3 subcellular localization in primary cultures of hippocampal neurons of CD mice was not significantly altered with respect to WT cells (F_1,77_ = 0.3922, *p* = 0.5331) (Fig 6E and S4A Fig).

**Fig 7:**
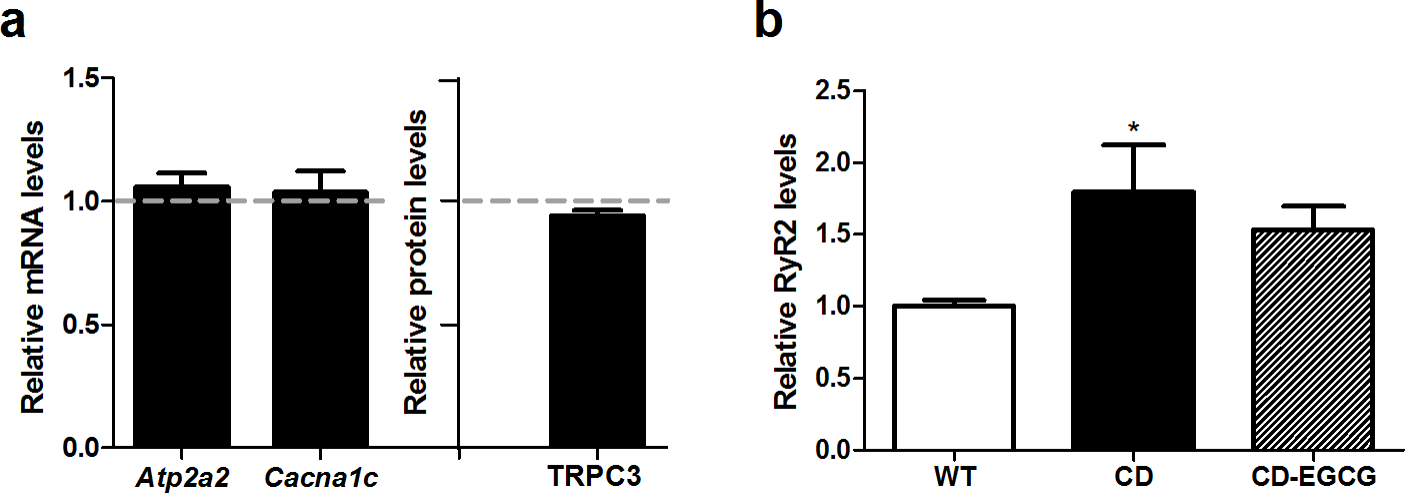
Impaired cellular calcium homeostasis in CD mice cardiac tissue. (a) Calcium-related gene (*Atp2a2*, *Cacna1c*) and protein (TRPC3) expression did not differ between WT and CD cardiac tissue/cells (*p* = 0.640, *p* = 0.158, *p* = 0.059, respectively). (b) Up-regulation of RyR2 was observed in left ventricles of CD mice with significant differences among groups (one-way ANOVA, F_2,16_ = 5.584, *p* = 0.014). n = 5–8 mice per group. *p* values are shown with asterisks (genotype) indicating values that are significantly different (One-way ANOVA with Bonferroni *post hoc* test). *p<0.05. White, WT; Black, CD. Plain, water; Striped, EGCG. Data are presented as the mean ± SEM.

In cardiac tissue, no differences in expression of *Cacna1c*, *Atp2a2* and TRPC3 were observed (Fig 7A), but interestingly, a significant higher amount of RyR2 was observed in CD mice when compared to WT mice (*p*<0.05, Bonferroni *post hoc* test), without treatment effect (Fig 7B).

### EGCG treatment normalizes nuclear levels of NRF2 in CD cardiomyocytes

**Fig 8:**
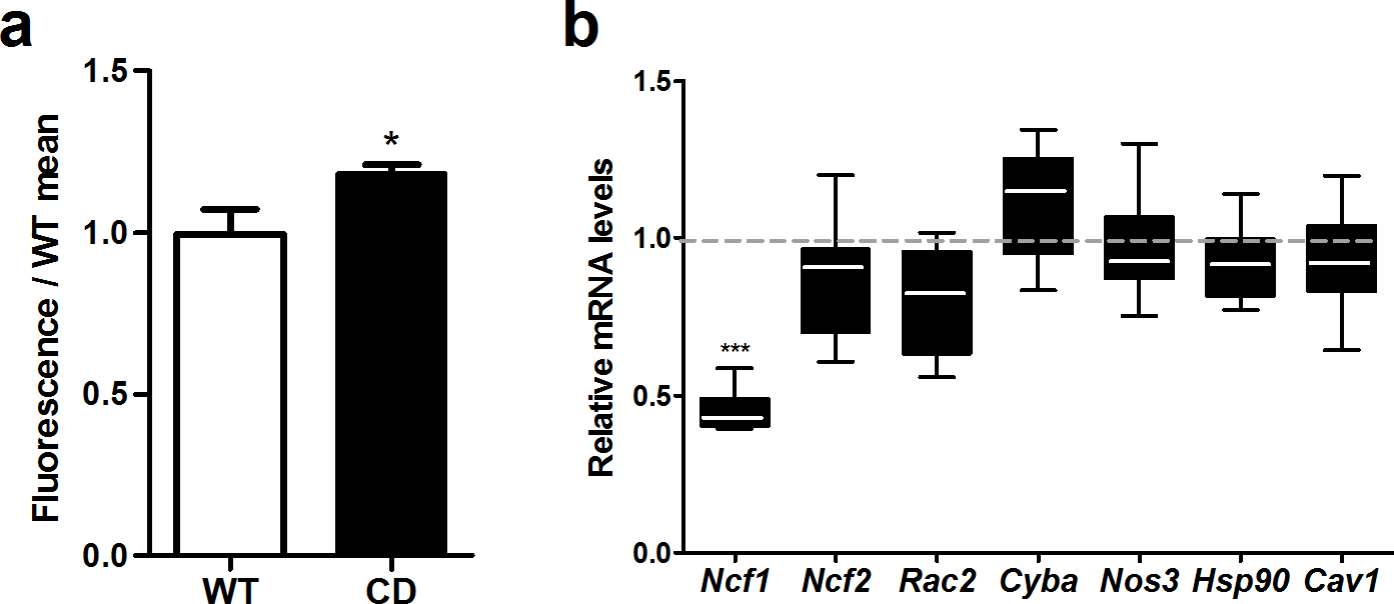
NADPH-oxidase and NO pathways were not affected by EGCG. (a) DHE staining in cardiac tissue showed a ≈20% increase in the general oxidative stress levels in CD mice (*p* = 0.04). (b) CD animals showed reduced expression levels of *Ncf1* (*p* = 0.0008) as previously described, but expression of other oxidative stress-related genes of the NADPH-oxidase and NO pathways was not affected. n = 3–4 mice per group. *p* values are shown with asterisks (genotype) indicating values that are significantly different (Unpaired *t*-test with Welch’s correction). *p<0.05; ***p<0.001. White, WT; Black, CD. Plain, water. Data are presented as the mean ± SEM.

Oxidative stress status, analyzed by DHE staining, showed a slight increase of around 20% (*p* = 0.04) in CD mice compared to WT mice (Fig 8A and S4B Fig). We investigated the expression levels of several genes related to the NADPH-oxidase complex or nitric oxide cascade. Reduced expression in heart mRNA levels of CD mice was observed for *Ncf1* (*t*_3_ = 14.15, *p* = 0.0008, unpaired *t-*test with Welch’s correction), but not for the other tested genes (*Cav1*, *Cyba*, *Hsp90*, *Ncf2*, *Nos3*, and *Rac2*) (Fig 8B) indicating that the mechanism by which the cardiac hypertrophy is improved does not involve these genes.

**Fig 9:**
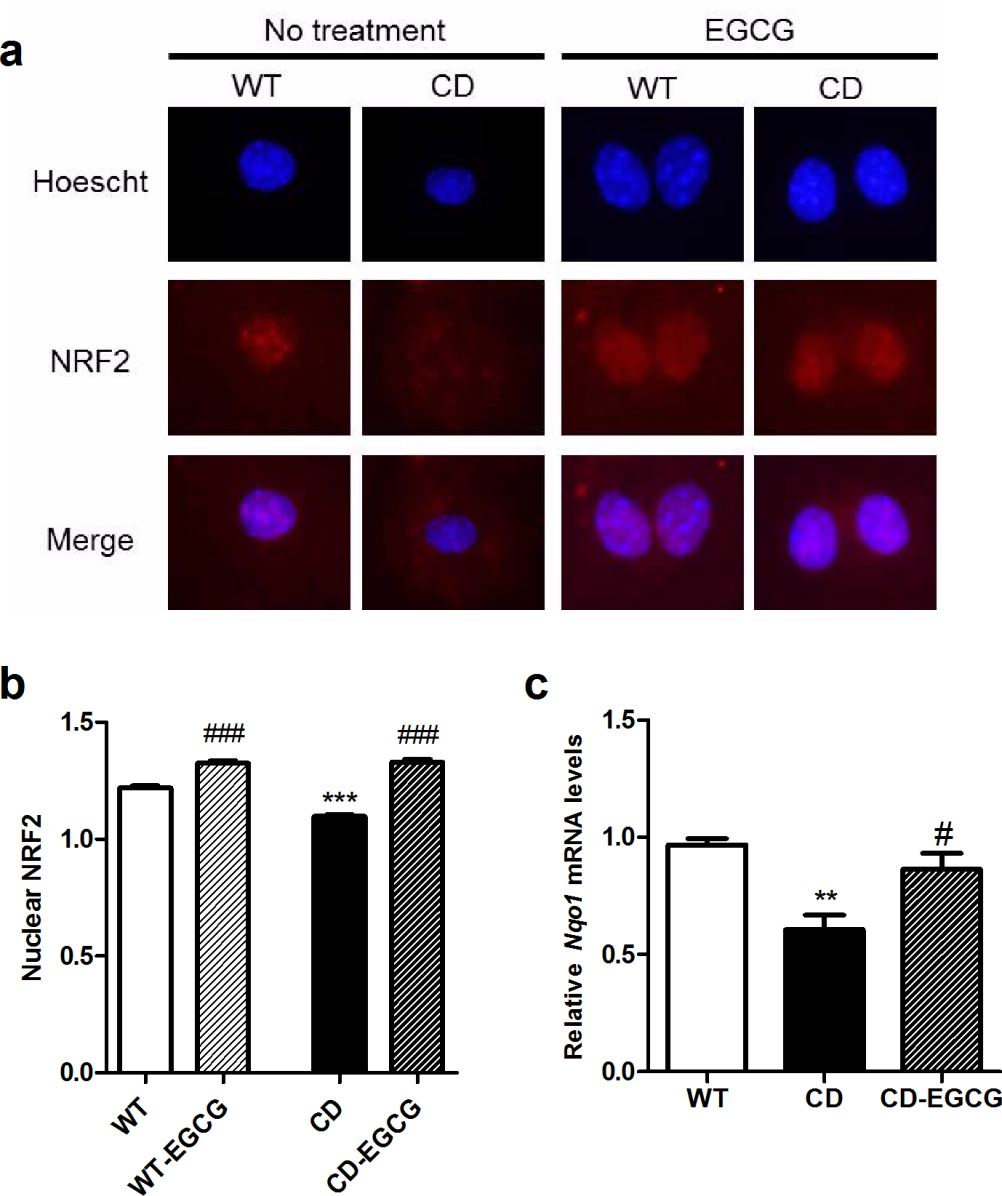
EGCG restores oxidative stress status via NRF2 pathway. (a) Representative images of cardiomyocytes cultures showed that NRF2 nuclear levels were decreased in CD cardiomyocytes in comparison with WT cells. After EGCG-treatment both genotypes increased the nuclear proportion of NRF2. Blue, DAPI; red, NRF2. (b) Histograms show the quantification of figures showed in (a). A clear interaction effect between genotype and treatment could be observed (F_1,756_ = 34,51, *p*<0.0001). n = 145–235 cardiomyocytes per group. (c) *Nqo1* expression levels were downregulated in CD cardiac tissue (*p*<0.01, Bonferroni post hoc test) and significantly higher after EGCG treatment (*p*<0.05, Bonferroni *post hoc* test). n = 4 mice/group. *p* values are shown with asterisks (genotype) or hashes (treatment) indicating values that are significantly different (two-way ANOVA or one-way ANOVA with Bonferroni *post hoc* test). *^,#^*p*<0.05, **^,##^*p*<0.01; ***^,###^*p*<0.001. White, WT; Black, CD. Plain, water; Striped, EGCG. Data are presented as the mean ± SEM.

Recent studies have revealed that NRF2 plays a role in Ang II induced oxidative stress and the subsequent hypertrophic remodeling of the heart [36], and that EGCG modulates the expression of the NRF2 regulated genes [37]. We observed that in basal culture conditions the amount of NRF2 in the nucleus of CD cardiomyocytes was significantly lower than in WT cells (*p*<0.001, Bonferroni *post hoc* test) (Fig 9A and 9B) in correlation with a decreased expression of NRF2 target genes as *Nqo1* (*p*<0.01, Bonferroni *post hoc* test) (Fig 9C). As expected, NRF2 levels in the cardiomyocytes’ nuclei of WT-EGCG treated cells were significantly higher than in untreated WT cells (*p*<0.001, Bonferroni *post hoc* test). NRF2 levels in the nucleus of CD EGCG-treated versus CD untreated cardiomyocytes were significantly different (*p*<0.001, Bonferroni *post hoc* test). CD-EGCG treated mice showed an increased expression of NRF2 target genes such as *Nqo1* compared to CD control mice (*p*<0.05, Bonferroni *post hoc* test) (Fig 9C). These results suggest a normalization of the oxidative stress-regulation pathways involving *Nrf2* via EGCG treatment.

## Discussion

Cardiovascular complications are the most common cause of health problems and even death in patients with WBS with 25–100 fold increased risk of sudden death compared to the age-matched normal population [9,38]. In addition, WBS individuals present cognitive, behavioral and neuropsychiatric problems that can affect their quality of life. In fact, the goal of independent living and competitive employment is not frequently achieved by adults with WBS [39]. Therefore, any potential treatment that might alleviate these phenotypes deserves to be studied in WBS.

Natural polyphenols extracts have attracted increasing attention since they have shown diverse biological activities and beneficial effects for an increasing number of chronic pathological conditions, including cardiovascular, cancer, inflammatory and neurological diseases [14,15]. For instance, administration of EGCG in different animal models of Down syndrome rescued hippocampal-dependent learning deficit [15,16]. Accordingly, administration of green tea ameliorated short-term memory deficits of CD mice when evaluated in the NOR test. In contrast with previous reports EGCG did not affect the performance of WT littermates in this test [15]. It should be noted, though, that EGCG diet did not rescue the spatial working memory deficits observed in CD animals in the spontaneous alternation test, pointing to specific effects of EGCG on short-term memory. Despite EGCG has been proposed as a therapeutic approach to treat anxiety [40,41], in our study we were not able to improve the performance of CD animals in anxiety-like behavior related behavioral tests. This apparently controversial result could be due to the different anxiety-related tests and EGCG dose used in each study, and/or to the different mechanisms of anxiety in each disorder. Finally, oral administration of EGCG, in the dose tested, does not have any effect on sociability.

EGCG has been shown to have a potent antioxidant activity being able to completely inhibit cardiac hypertrophy and improve cardiac performance in pressure overloaded hearts [42,43]. In WBS patients and mice with elastin deficiency [13,30], the p47phox subunit of the NADPH oxidase (NOX) encoded by *NCF1*, has been found to modulate blood pressure and the cardiovascular phenotype. CD mice show a milder cardiovascular phenotype than other elastin deficient mice that correlates with reduced levels of *Ncf1* expression, reinforcing the idea that the control of NOX activity and oxidative stress may help to diminish the cardiac affectation [10]. Oxidative stress status, analyzed by DHE staining, showed slight differences (around 20%) between WT and CD mice. In accordance with previous results [10], *Ncf1* was significantly reduced in hearts of CD mice. However, we found a significant reduction in the nuclear NRF2 levels of CD cardiomyocytes that correlates with lower mRNA expression of target genes, like *Nqo1*. We show that EGCG treatment in CD mice is effective to improve the cardiac hypertrophy by recovering the cardiomyocytes’ normal size. EGCG treatment also restored nuclear levels of NRF2 in correlation with normalization of mRNA expression of target genes. CD mice also had up-regulated levels of RyR2, the major molecule responsible for Ca^2+^-mediated myofilament contraction. Upregulation of RyR2 can alter channel stoichiometry with detrimental consequences for cardiomyocytes function and increased Ca^2+^ sensitivity of cardiac myofilaments [44], such as in the dilated cardiomyopathy secondary to anthracyclines treatment [45]. EGCG has been proved to decrease calcium sensitivity in cardiac myofilaments becoming a promising therapeutic agent for the hypertrophic cardiomyopathy caused by increased Ca^2+^ sensitivity [21]. Thus, the improvement of cardiac hypertrophy by EGCG in CD mice could involve both mechanisms. On one hand, changes in the oxidative stress regulation by NRF2 would protect against angiotensin II-induced hypertrophic remodeling, as previously reported [36]. On the other hand, decreasing Ca^2+^ sensitivity due to RyR2 upregulation without affecting the amount of RyR2 would also have a protective effect for the heart.

The beneficial effects of EGCG treatment in the neurocognitive phenotype of CD mice were restricted to improvements in short-term memory with no effects on spatial working memory, anxiety or sociability. Several studies have supported the role of EGCG in the expression or activity of BDNF [16,25]. CD animals fed with EGCG presented normalized expression levels of *Bdnf* without any effect in neuroanatomical remodeling. It has been shown that the membrane localization of TRPC3 channels, important mediators of BDNF-mediated Ca^2+^ signal generation and dendritic remodeling [46], is regulated by general transcription factor TFII-I encoded by *GTF2I* deleted in WBS[33,34]. In hippocampal primary cultures of CD mice we observed altered intracellular calcium flux although no significant changes in TRPC3 distribution were observed. It has been shown that protective effects of EGCG on spine formation and maturation only occur within a certain dose range: 10 and 25mg/kg of EGCG reversed spine damage but not a 50mg/kg dose [47]. The higher dose of EGCG used in our study (125mg/kg) has been proved to improve cognition in other genetic models and also caused a reduction in the brain weight of treated WT animals [15,16]. Therefore, the dose used in this study could also explain the lack of effects on neuroarchitecture in CD mice or even the deleterious effect of EGCG in WT mice.

Abnormalities in calcium homeostasis have been frequently described in WBS. These include hypercalcaemia, which usually occurs during the first few years of life (most cases resolving by four years of age), and hypercalciuria, that may persist longer [48]. Memory processing and cardiomyocytes function requires tightly controlled signaling cascades, many of which are dependent upon intracellular calcium (Ca^2+^). We propose that altered calcium homeostasis in WBS patients, without being the major player, plays a relevant role in the cognitive and cardiovascular phenotype of these patients. The altered calcium homeostasis in WBS patients could be due to upregulated expression of ryanodine receptors and increased calcium sensitivity of neurons and cardiomyocytes. Therefore, EGCG may be a promising therapeutic agent for WBS individuals, among other mechanisms, by decreasing calcium sensitivity, increasing expression of NRF2-dependent endogenous antioxidants and increasing BDNF expression levels.

Nevertheless, future research should be focused on the deep mechanism of EGCG, since despite encouraging results of this molecule in preclinical and clinical studies there are controversial results in some studies, probably due to differences in the dose, duration of treatment or route of administration of EGCG.

## Supporting information

S1 Fig Epigallocatechin-3-gallate (EGCG) intake. (a) Body weights of CD mice were lower when compared with WT mice without influence of treatment (F_3,39_ = 8.361, *p* = 0.0002). (b) Daily EGCG consumption (ml per day) changed over time (repeated measures ANOVA, F_11_ = 4.893, *p*<0.001) but this significant effect is mainly due to the high uptake for day 2 (Bonferroni *post hoc* test); there were no significant differences between consumption of EGCG or water (main effect of treatment F_1,12_ = 0.049, *p* = 1.0).
(TIF)

S2 Fig Hippocampal architecture and spine density. (a) Representative 1360x1024 images of hippocampus obtained with an Olympus DP71 camera attached to an Olympus BX51 microscopy with an Olympus U-RFL-T source of fluorescence at 4x magnification. (b) Representative 1024x1024 images of apical dendrites obtained in a TCS SP2 LEICA confocal microscopy at 60X magnification.
Pyramidal neurons were labelled in green due to transgenic expression of Thy1-YFP [22].
(TIF)

S3 Fig Time to complete memory test. (a) Representation of the preference index in the training session. Both genotypes equally explored the two identical objects (A+A). n = 8–9 per genotype. Grey bar, familiar object (A). (b) Total exploration time (time exploring the objects in the training session and in the test session) was measured. A two-way ANOVA indicated a significant effect of genotype (F_1,26_ = 12.68, *p* = 0.0015) but no effect of treatment (F_1,26_ = 0.06951, *p* = 0.7941). n = 6–9 per genotype. (c) Total time to complete 15 trials in the spontaneous alternation test. A two-way ANOVA indicated no differences in genotype (F_1,39_ = 0.3587, *p* = 0.5527) or treatment (F_1,39_ = 0.4723, *p* = 0.4960). n = 8–13 per genotype. *p* values are shown with asterisks indicating values that are significantly different in a two-way ANOVA with Bonferroni *post hoc* test (*p<0.05, genotype effect). White, WT; Black, CD. Data are presented as the mean ± SEM.
(TIF)

S4 Fig Immunofluorescences analysis. (a) Expression of TRPC3 (green) in primary hippocampal neurons. For each neuron, expression of TRPC3 was normalized to MAP2 staining (red). (b) Qualitative examination of superoxide levels in left ventricular sections stained with DHE (red). 1360x1024 images were obtained with an Olympus DP71 camera attached to an Olympus BX51 microscopy with an Olympus U-RFL-T source of fluorescence (40X for neural cultures, 20X for left ventricle).
(TIF)

S1 Table Primer sequences used in qPCR/semiquantitative PCR. (PDF)
