# Supplementary figures and images for: Epigallocatechin-3-gallate improves cardiac hypertrophy and short-term memory deficits in a Williams-Beuren syndrome mouse model
Source: PLoS One. 2018 Mar 19;13(3):e0194476. doi: 10.1371/journal.pone.0194476 (PMC5858783; doi:10.1371/journal.pone.0194476)

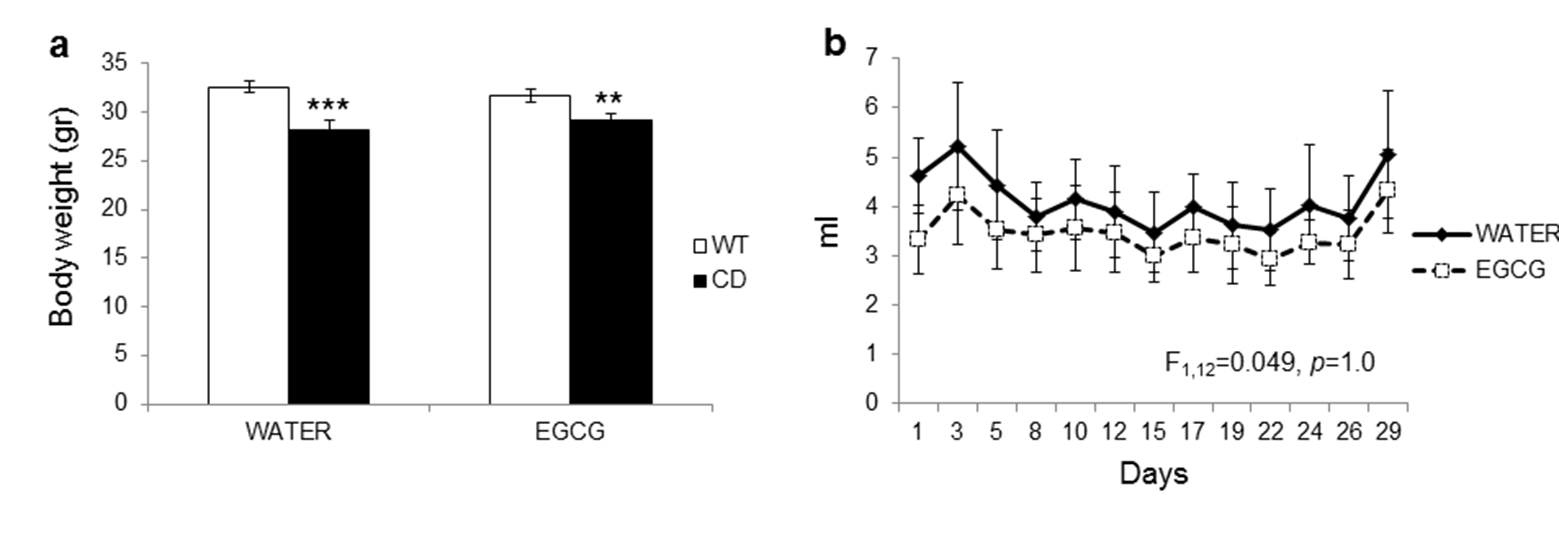

Supplement: S1 Fig — (a) Body weights of CD mice were lower when compared with WT mice without influence of treatment (F3,39 = 8.361, p = 0.0002). (b) Daily EGCG consumption (ml per day) changed over time (repeated measures ANOVA, F11 = 4.893, p<0.001) but this significant effect is mainly due to the high uptake for day 2 (Bonferroni post hoc test); there were no significant differences between consumption of EGCG or water (main effect of treatment F1,12 = 0.049, p = 1.0). (TIF) [file pone.0194476.s001.tif]

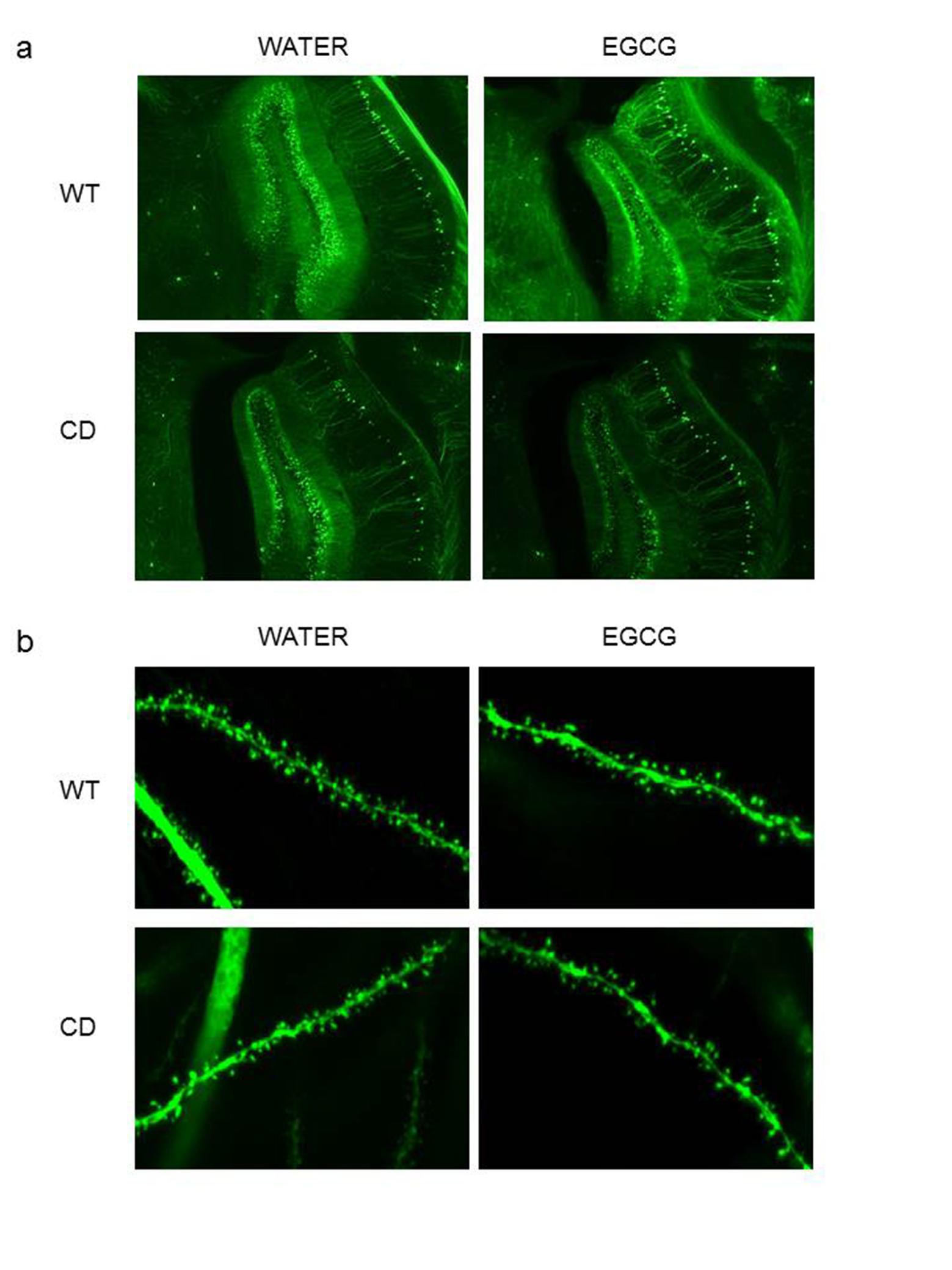

Supplement: S2 Fig — (a) Representative 1360x1024 images of hippocampus obtained with an Olympus DP71 camera attached to an Olympus BX51 microscopy with an Olympus U-RFL-T source of fluorescence at 4x magnification. (b) Representative 1024x1024 images of apical dendrites obtained in a TCS SP2 LEICA confocal microscopy at 60X magnification. Pyramidal neurons were labelled in green due to transgenic expression of Thy1-YFP [22]. (TIF) [file pone.0194476.s002.tif]

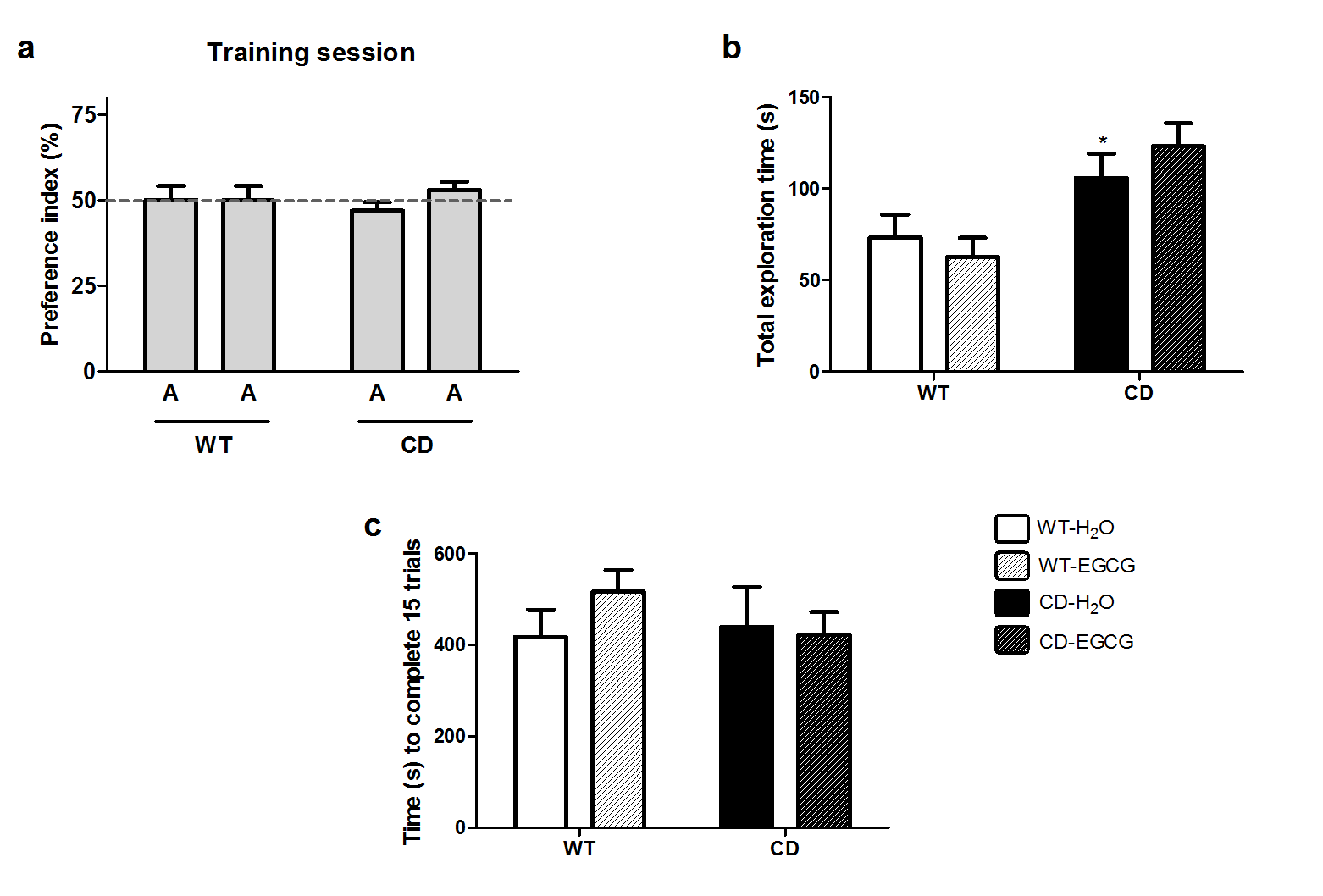

Supplement: S3 Fig — (a) Representation of the preference index in the training session. Both genotypes equally explored the two identical objects (A+A). n = 8–9 per genotype. Grey bar, familiar object (A). (b) Total exploration time (time exploring the objects in the training session and in the test session) was measured. A two-way ANOVA indicated a significant effect of genotype (F1,26 = 12.68, p = 0.0015) but no effect of treatment (F1,26 = 0.06951, p = 0.7941). n = 6–9 per genotype. (c) Total time to complete 15 trials in the spontaneous alternation test. A two-way ANOVA indicated no differences in genotype (F1,39 = 0.3587, p = 0.5527) or treatment (F1,39 = 0.4723, p = 0.4960). n = 8–13 per genotype. p values are shown with asterisks indicating values that are significantly different in a two-way ANOVA with Bonferroni post hoc test (*p<0.05, genotype effect). White, WT; Black, CD. Data are presented as the mean ± SEM. (TIF) [file pone.0194476.s003.tif]

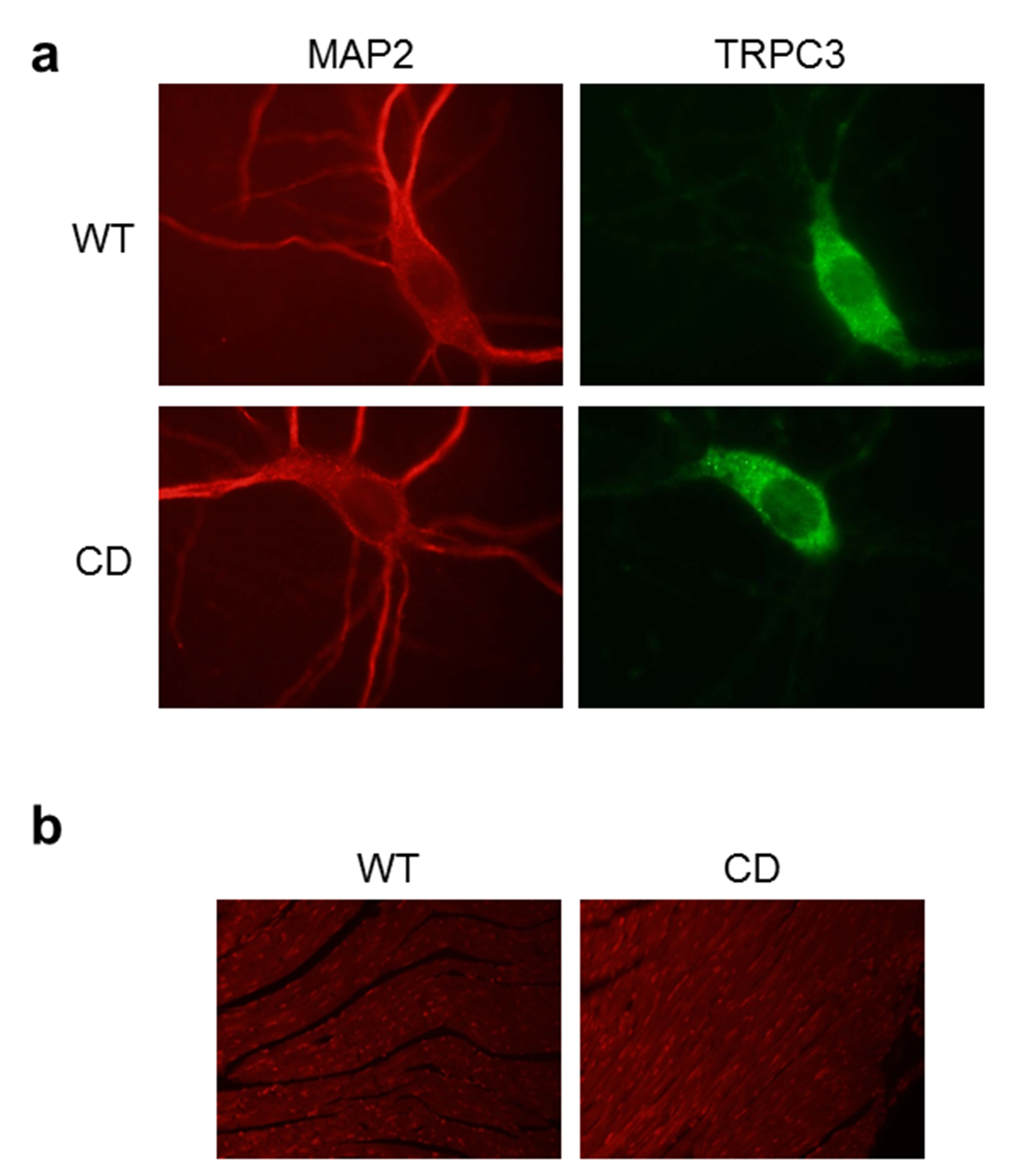

Supplement: S4 Fig — (a) Expression of TRPC3 (green) in primary hippocampal neurons. For each neuron, expression of TRPC3 was normalized to MAP2 staining (red). (b) Qualitative examination of superoxide levels in left ventricular sections stained with DHE (red). 1360x1024 images were obtained with an Olympus DP71 camera attached to an Olympus BX51 microscopy with an Olympus U-RFL-T source of fluorescence (40X for neural cultures, 20X for left ventricle). (TIF) [file pone.0194476.s004.tif]
