# Supplementary material for: Epigallocatechin-3-gallate improves cardiac hypertrophy and short-term memory deficits in a Williams-Beuren syndrome mouse model
Source: PLoS One. 2018 Mar 19;13(3):e0194476. doi: 10.1371/journal.pone.0194476 (PMC5858783; doi:10.1371/journal.pone.0194476)
Supplement: S1 Table — (PDF) [file pone.0194476.s005.pdf]

Supplementary table 1. Primer sequences used in qPCR/semiquantitative PCR

Gene		Sequence (5' →3')	Size	Location	Tm (ºC)
Bdnf	L	AGTCTCCAGGACAGCAAAGC	189	exon 1	63.6
	R	ATTCACGCTCTCCAGAGTCC		exon 2	63.4
Pik3r1	L	TGGCTGGGGAATGAAAATAC	179	exon 8	63.5
	R	CAGCCCTGCTTACTGCTCTC		exon 9	64.2
Ncf1	L	ATACTTCAACGGCCTCATGG	233	exon 4	59.96
	R	CTGTTCCCGAACTCTTCTCG		exon 6	59.98
Ncf2	L	CCGACAAGAAGGACTGGAAG	219	exon 1	59.84
	R	CAAGGTCGTACTTCTCCATTCTG		exon 2-3	60.17
Rac2	L	AACGCCTTCCCTGGAGAATA	150	exon 2	60.95
	R	TGTCTGTGGGTAGGAGAGTGG		exon 3	60.16
Cyba	L	AGATCGAGTGGGCCATGT	102	exon 1	59
	R	ACCACTGTGTGAAACGTCCA		exon 2	60
Nos3	L	TCCTGTGCATGGATGAGTATG	157	exon 13	63.6
	R	CTAGGGGAGCTGTTGTACGG		exon 14	63.5
Hsp90	L	GGAGGAGAGCAAGGCAAAGT	161	exon 9	64.7
	R	TCATGATCCGTTCCATGTTG		exon 10	64.5
Cav1	L	GCAGACGAGGTGACTGAGAA	184	exon 3	63.6
	R	CAGTGAAGGTGGTGAAGCTG		exon 4	63.6
Cacna1c	L	CAGGAAGATGAACACCGACA	164	exon 44	63.8
	R	TGAGAGATGTCTCCCCCTTG		exon 45	64.3
Atp2a2	L	TTCCGTTACCTGGCTATTGG	153	exon 16	63.6
	R	TCCATCGAAGTCTGGGTTGT		exon 18	64.5
Nqo1	L	GGCTGGTTTGAGAGAGTGCT	180	exon 4	63.6
	R	ACTCTGAATCGGCCAGAGAA		exon 6	63.9
Limk1	L	CCTACCTCCATTCGATGAACA	262	exon 12	63.81
	R	CCAAAGGAAAACACGTCCA		exon 14	64.35
Rps28	L	TAGGGTAACCAAAGTGCTGGG	103	exon 1-2	67.9
	R	GACATTTCGGATGATAGAGCGG		exon 3	66.6
